# A Tri-Source Microbiome Dataset from Human Sleep Environments: Saliva, Pillow Dust, and Bedroom Air

**DOI:** 10.1159/lfg/adlag001

**Published:** 2026-08-18

**Authors:** Jeong Yun Jang, Jaeha Lee, Hyunjun Yun, Jinho Yang

**Affiliations:** a Department of Radiation Oncology, Konkuk University Medical Center, Konkuk University School of Medicine, Seoul, Republic of Korea; b Department of Radiation Oncology, Asan Medical Center, University of Ulsan College of Medicine, Seoul, Republic of Korea; c The AI Convergence Appliance Research Center, Korea Electronics Technology Institute, Gwangju, Republic of Korea; d Department of Occupational Health and Safety, Semyung University, Jecheon, Republic of Korea

**Keywords:** Microbiome, Indoor air, Saliva, Pillow dust, Sleep environment

## Abstract

**Introduction:**

Despite the recognized importance of the human microbiome, integrated microbial exposures within sleep environments remain poorly understood. Sleep represents a prolonged period of close contact with indoor microbial reservoirs, yet the connectivity between environmental sources and the human oral microbiome has not been comprehensively examined.

**Methods:**

We conducted a pilot study involving eight healthy adults, collecting paired saliva, pillow dust, and bedroom air samples under standardized conditions. Microbial communities were characterized using 16S rRNA gene amplicon sequencing targeting the V3–V4 region. Sequence data were processed using Cutadapt for primer removal and DADA2 for amplicon sequence variant inference, followed by taxonomic assignment and diversity analyses.

**Results:**

Pillow dust exhibited significantly higher alpha diversity, including species richness and phylogenetic diversity, compared to saliva and air (*p* < 0.05). Beta diversity analysis revealed distinct clustering by sample type, with pillow dust and air showing greater similarity to each other than to saliva. At the genus level, significant correlations were observed between air and pillow dust (e.g., *Brevitalea*, *Gardnerella*) and between pillow dust and saliva (e.g., *Actinomyces*, *Bifidobacterium*). These patterns suggest potential microbial transfer pathways mediated by gravitational settling and direct physical contact. Given the small sample size, findings should be interpreted as exploratory.

**Conclusion:**

This study provides a novel tri-source microbiome dataset linking human and environmental microbial communities in real-world sleep settings. The results highlight the sleep environment as a relevant exposomic factor influencing oral microbiome dynamics and support further large-scale investigations into host-environment microbial interactions.

## Introduction

In recent years, interest in the human microbiome has significantly increased across various scientific disciplines [1, 2]. Numerous studies have demonstrated a strong association between the human microbiome and overall health [3, 4]. Dysbiosis of the microbiome has been implicated in a wide range of diseases, including cancer, neurological disorders, respiratory illnesses, gastrointestinal diseases, and cardiovascular conditions [5–10]. As a result, there has been a growing effort to explore the human microbiome for potential applications in diagnostics and therapeutics [11, 12].

To date, most human microbiome studies have primarily focused on fecal samples, which are considered a representative source of gut microbiota [13, 14]. However, recent findings suggest that the salivary microbiome also plays a critical role in human health [15–18]. In particular, respiratory diseases, periodontal disorders, and gastrointestinal illnesses have shown strong correlations with alterations in the salivary microbiome, indicating its potential as a non-invasive biomarker for disease detection and monitoring [19–21].

In parallel, a growing body of research has characterized indoor airborne and dust-associated microbiomes, demonstrating that indoor air and household dust harbor diverse microbial communities shaped by outdoor inputs, building characteristics, and human occupancy, including skin and oral microbial shedding [22–24]. These studies suggest that indoor dust and air can act as reservoirs or archives of human–environment microbial exchange within residential spaces [22]. However, most prior work has examined indoor air, settled dust, or human microbiomes separately, without simultaneously profiling multiple, closely related microhabitats.

Moreover, the salivary microbiome is influenced not only by host factors but also by environmental exposures [25, 26]. Airborne pollutants such as particulate matter, volatile organic compounds, and microbial aerosols can interact with the oral cavity, potentially altering the composition of the salivary microbiota [27–29]. Given that airborne microorganisms may be inhaled or deposited during sleep, their contribution to the salivary microbiome warrants investigation. Despite this, no previous study has directly examined the relationship between the salivary microbiome and the indoor airborne microbiome.

This study collected and analyzed saliva, indoor air near the sleeping area, and pillow surface dust from the same individuals to investigate microbial similarities across these sample types. This approach provides insight into how environmental microbiomes in the sleep environment may relate to the human oral microbiome.

## Methods

### Study Design

This study, the first to investigate this specific relationship, aimed to examine the microbiome connectivity among saliva, pillow surface dust, and indoor air within the human sleep environment. A total of eight healthy adults were enrolled, and one saliva, one pillow dust, and one bedroom air sample were collected from each participant, yielding 8 saliva, 8 pillow dust, and 7 air samples after quality control (one air sample was excluded due to insufficient DNA for sequencing). As illustrated in Figure 1, all three sample types were collected simultaneously from each participant to ensure temporal and spatial consistency. Samples were immediately stored at −80°C prior to analysis. Metagenomic profiling targeting the V3–V4 region of the 16S rRNA gene was then performed to characterize microbial communities across sources. To minimize contamination, standardized protocols and identical equipment were used for environmental sampling across all participants, and all samples were handled using sterile tools and standardized contamination-minimizing procedures and were rapidly frozen after collection. Additionally, as this work was designed as an exploratory pilot study, we included field and extraction blanks to monitor background contamination.

**Fig. 1.**
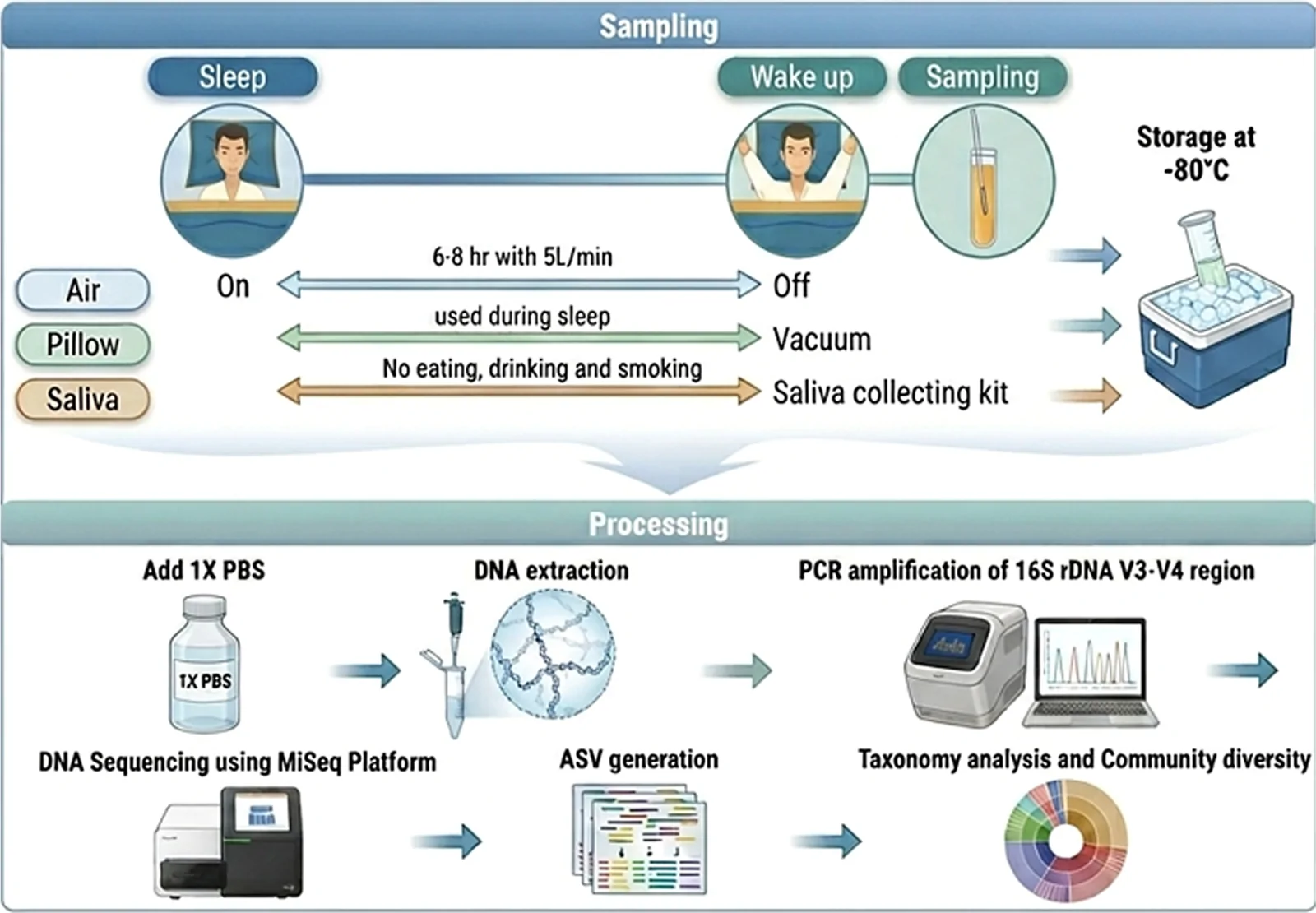
Integrated sampling and analysis workflow of tri-source microbiome profiling in the human sleep environment.

### Sample Collection Process

Healthy individuals were recruited between March and August 2025 to collect human saliva samples. Subjects lived in Chungcheongbuk-do, South Korea. Participants provided samples using a saliva collection kit PDX-026 (PDXen Biosystem Inc., Yongin-si, South Korea). The criteria for healthy individuals included an age range of 20–40 years, no history of cancer, no dental cavities, and no use of medications for diseases such as diabetes or hypertension. This study included eight participants (6 females and 2 males; 2 smokers and 6 non-smokers) aged 20–36 years, all residing in Chungcheongbuk-do, Republic of Korea. To minimize contamination, participants collected saliva samples immediately upon waking, without eating or drinking during sleep.

Air samples were collected by trained research personnel using a 37 mm gelatin membrane filter (Sartorius, Germany) operated at a constant airflow of 5 L/min for 6–8 h during sleep, with the sampler positioned at a height of 1.5 m near the participant’s bed. During air sampling, all windows were kept closed, and any air purifiers were turned off to reduce uncontrolled air exchange. Filters were handled exclusively with sterilized tweezers, transferred into sterile Petri dishes or containers immediately after sampling, transported on ice, and stored at −80°C until further processing.

Pillow dust samples were collected from the pillow used during sleep immediately upon waking, using the same vacuum and filter system for all participants. A standardized pillow surface area (0.3 m × 0.3 m) was vacuumed for 1 min at a flow rate of 1,000 L/min to ensure comparability across individuals. The vacuum filter and accessories were sterilized before each sampling session, and filters were handled with sterilized tweezers after sampling. Pillow dust filters were placed in sterile containers, transported on ice, and stored at −80°C until DNA extraction.

### DNA Extraction

Pillow dust and air filter samples were mixed with 10 mL of 1X PBS (Phosphate-Buffered Saline) and incubated for 24 h at 4°C after vortexing. [29]. Subsequently, 300 µL of saliva, pillow dust, and airborne filter samples were added to a 96-well bead-beating plate. DNA was extracted from both the pellet and supernatant using the DNeasy PowerSoil Pro kit (QIAGEN, Germany). The extracted DNA was stored at −20°C until further processing.

### Library Construction and Sequencing

The sequencing libraries are prepared using the Illumina 16S Metagenomic Sequencing Library protocols to amplify the target region. The input gDNA 5 ng was PCR amplified with 5× reaction buffer, 1 mm of dNTP mix, 500 nm each of the universal F/R PCR primer, and Herculase II fusion DNA polymerase (Agilent Technologies, Santa Clara, CA). The cycle condition for the 1st PCR was 3 min at 95°C for heat activation and 25 cycles of 30 s at 95°C, 30 s at 55°C, and 30 s at 72°C, followed by a 5-min final extension at 72°C. The universal primer pair with Illumina adapter overhang sequences used for the first amplifications was as follows: PCR Forward Primer 5' (TCGTCGGCAGCGTCAGATGTGTATAAGAGACAG-[locus specific sequence]) and PCR Reverse Primer 5' (GTCTCGTGGGCTCGGAGATGTGTATAAGAGACAG-[locus specific sequence])

The 1st PCR product was purified with AMPure beads (Agencourt Bioscience, Beverly, MA). Following purification, the 10 µL of 1st PCR product was PCR amplified for final library construction containing the index using NexteraXT Indexed Primer. The cycle condition for the 2nd PCR was the same as the 1st PCR condition except for 10 cycles. The PCR product was purified with AMPure beads. The final purified product is then quantified using VICTOR Nivo™ (Perkin Elmer) with PicoGreen reagents and qualified using the TapeStation D1000 ScreenTape (Agilent Technologies, Waldbronn, Germany). The libraries are normalized, pooled, and quantified using qPCR following the qPCR Quantification Protocol Guide (KAPA Library Quantification Kits for Illumina Sequencing Platforms). Then we sequenced using the MiSeq™ platform (Illumina, San Diego, USA).

DNA yields from the blanks were below the threshold required for library preparation and sequencing, and thus, these samples were not included in downstream community analyses. This limitation is acknowledged, and the observed microbial profiles are interpreted with caution, while the negligible DNA in blanks suggests that the detected communities predominantly reflect the sampled matrices. Additionally, one sample (ID: DU006) was excluded from downstream analysis due to an inadequate quantity of genomic DNA for sequencing library preparation (online suppl. Table S1; for all online suppl. material, see https://doi.org/10.1159/lfg/adlag001).

### Data Preprocessing and Amplicon Sequence Variant Generation

After sequencing, Cutadapt (v3.2) was utilized to remove adapter and primer sequences from the raw data. Additionally, forward and reverse reads were trimmed to 250 bp and 200 bp using Cutadapt (v3.2) [30]. To generate amplicon sequence variant (ASV) sequences, the reads underwent error correction, merging, and denoising processes with DADA2 (v1.18.0) [31]. Sequences with an expected error of two or more were excluded. Erroneous reads were denoised based on an established error model. Following error correction, paired-end reads were merged by overlapping. Chimeric sequences were eliminated using the consensus method with the remove BimeraDenovo function in DADA2. ASVs with lengths shorter than 350 bp were filtered out using R (v4.0.3). Moreover, for microbial community comparison analysis, normalization was performed using QIIME (v1.9) [32]. During this process, subsampling was conducted based on the sample with the lowest read count to ensure comparability between samples. The resulting ASVs were utilized for downstream analysis. In this workflow, all upstream sequence processing, including quality filtering, denoising, chimera removal, and ASV inference, was completed in DADA2. The resulting ASV table was exported as a BIOM file in R and imported into QIIME v1.9.1 solely for normalization and diversity analyses, while retaining the DADA2-inferred ASVs as features without further clustering.

### Microbial Community Characterization

To assign taxonomy, each ASV was aligned to the organism with the highest similarity in the corresponding reference database (NCBI_16S), using algorithms such as Bayesian classifier (DADA2_v1.18.0, confidence value: 50) [33]. On average, 80.7% of reads and 66.56% of ASVs were assigned at the species level using this confidence threshold; however, given the known limitations of the V3–V4 region for precise species-level discrimination, these assignments were interpreted cautiously, and our main biological interpretations focused on genus-level patterns. QIIME (v1.9.1) was utilized for downstream ASV-based diversity analyses using the BIOM-formatted ASV table generated by DADA2, while retaining the DADA2-inferred ASVs as features without further clustering.

Alpha diversity metrics, including the observed ASVs, Shannon index, and Gini-Simpson index, were calculated to assess microbial complexity within each sample. observed ASVs, reflecting species richness, were also included. Multiple sequence alignment and phylogenetic tree construction were performed using MAFFT (v7.475) [34] and FastTreeMP (v2.1.10) [35], respectively. Beta diversity, representing inter-sample community dissimilarity, was assessed using Bray-Curtis, weighted UniFrac, and unweighted UniFrac distances. The genetic relationship among samples was visualized based on principal coordinate analysis (PCoA) and a UPGMA tree.

All statistical analyses were performed using non-parametric methods due to the nonnormal distribution of microbiome data. Alpha diversity indices were compared among air, pillow, and saliva samples using the Kruskal-Wallis test, followed by Dunn’s post hoc test with Bonferroni correction for multiple comparisons. To test for significant differences in community composition among groups, permutational multivariate analysis of variance (PERMANOVA) was conducted using 9,999 permutations, with pseudo-F statistics and R^2^ values reported. To assess whether significant PERMANOVA results were driven by differences in group dispersion rather than centroid location, a permutational analysis of multivariate dispersions was performed with 9,999 permutations, where distances from individual samples to their group centroid were compared among groups using an *F*-test. All beta diversity analyses were performed using PCoA-derived coordinates across all principal coordinate axes. Differences in taxonomic composition at the genus and species levels were evaluated using the Kruskal-Wallis test. Pairwise associations between microbial taxa in air, pillow, and saliva samples were examined using Spearman’s rank correlation analysis. All statistical analyses were conducted using R software (version 4.5.2). A *p* value <0.05 was considered statistically significant.

## Results

### Community Diversity

Alpha diversity, including observed ASVs, Shannon index, and Gini-Simpson index, was significantly higher in pillow samples than in both air and saliva (*p* < 0.05). Saliva samples also showed significantly higher ASV richness, Shannon index, and Gini-Simpson index compared with air samples (*p* < 0.05) (Fig. 2a–c). These findings indicate that the pillow microbiome exhibits greater species richness and evenness, as well as higher phylogenetic diversity, than those observed in air and saliva, suggesting that pillows may act as a more diverse and stable microbial reservoir. In contrast, air samples displayed the lowest alpha diversity, indicating relatively limited microbial complexity.

**Fig. 2.**
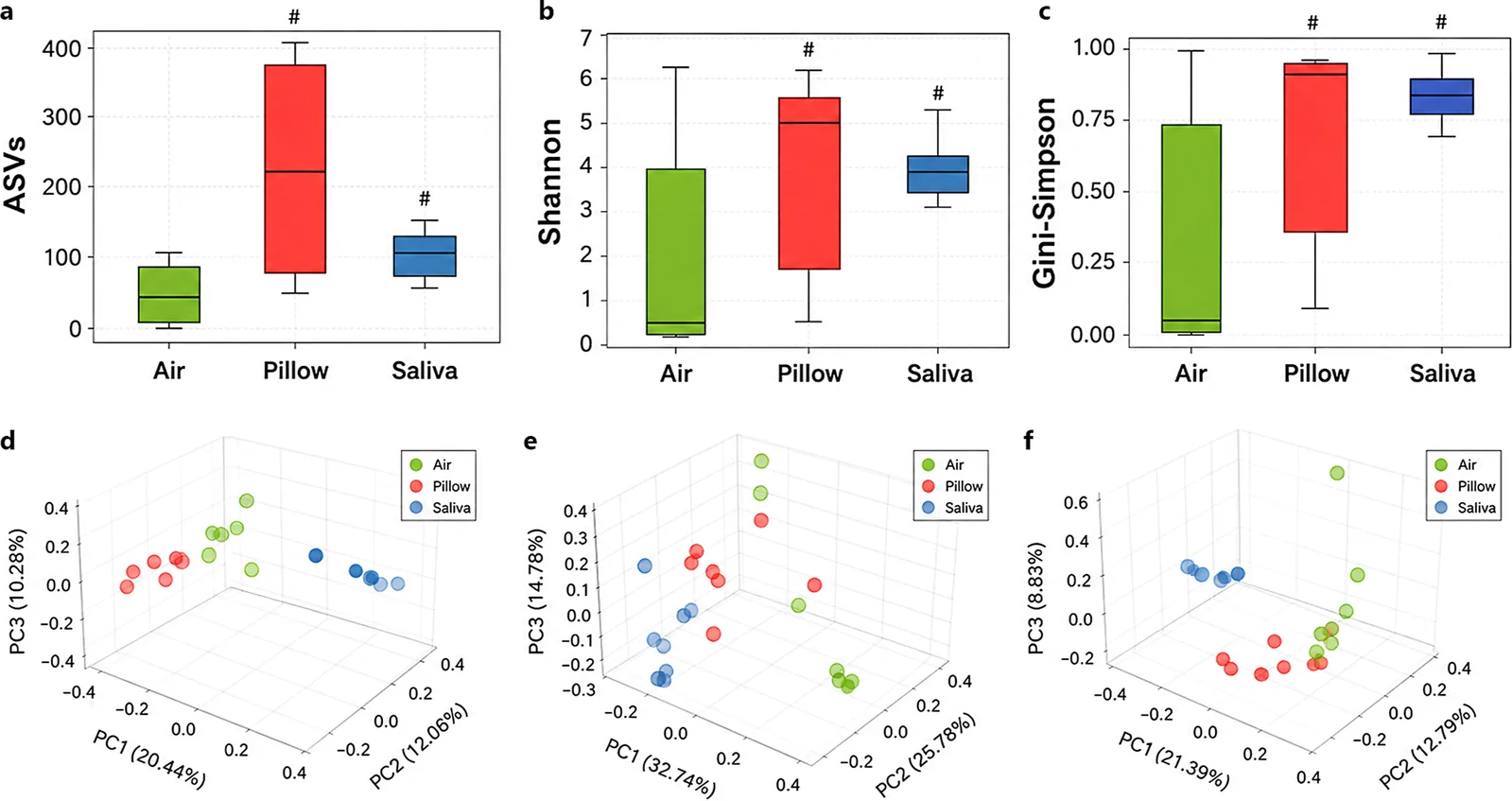
Diversity of microbial communities across saliva, pillow dust, and indoor air samples. Alpha-diversity: observed ASVs (**a**), Shannon index (**b**), and Gini-Simpson index (**c**). # indicates a statistically significant difference compared with Air (*p* < 0.05). Beta-diversity: Principal coordinate analysis (PCoA) based on Bray-Curtis (**d**), weighted UniFrac (**e**), and unweighted UniFrac (**f**) distances.

Across PCoA plots of beta diversity based on Bray-Curtis, weighted UniFrac, and unweighted UniFrac distances, samples clustered primarily by sample type, indicating distinct microbial community structures among the three groups (Fig. 2d–f). These compositional differences were statistically significant when tested by sample type (PERMANOVA: weighted UniFrac, R^2^ = 0.334 and *p* = 0.0001; Bray-Curtis, R^2^ = 0.286 and *p* = 0.0001; unweighted UniFrac, R^2^ = 0.293 and *p* = 0.0001). In addition, multivariate dispersion differed significantly among sample types (permutational analysis of multivariate dispersion: weighted UniFrac, F = 5.70 and *p* = 0.012; Bray-Curtis, F = 15.85 and *p* = 0.0006; unweighted UniFrac, F = 20.48 and *p* = 0.0001), indicating that both between-group separation and within-group variability contributed to the observed beta-diversity patterns. Notably, the microbial communities of air and pillow dust samples showed relatively shorter intergroup distances, suggesting greater similarity between these two environments. In contrast, saliva samples formed more compact and tightly clustered groups, indicating lower within-group variability and a more homogeneous microbial community structure than environmental samples.

### Metagenomic Analysis

Figure 3 shows the dominant genera across pillow dust, indoor air, and saliva samples, where dominant genera were defined as those with a mean relative abundance of ≥1% in at least one sample type. In pillow samples, the most abundant genera were *Pseudomonas* (24.0 ± 41.2%), *Staphylococcus* (18.6 ± 22.5%), *Bacillus* (8.8 ± 23.2%), *Lactobacillus* (8.1 ± 10.0%), *Corynebacterium* (6.4 ± 5.7%), and *Cutibacterium* (5.2 ± 6.8%). Air samples were dominated by *Rhodococcus* (28.5 ± 45.1%), *Rhodococcoides* (28.0 ± 44.2%), and *Caballeronia* (13.6 ± 33.3%), whereas saliva samples were primarily composed of *Streptococcus* (53.8 ± 16.3%), *Haemophilus* (15.9 ± 13.2%), and *Rothia* (7.7 ± 6.7%). Among all sample types, *Streptococcus*, *Corynebacterium*, and *Rothia* were consistently detected with mean relative abundances greater than 0.15%. Previous studies have reported that *Streptococcus* and *Corynebacterium* are ubiquitous bacteria distributed across diverse environments, including soil, water, and plants, and are also common members of the human microbiome, particularly on the skin, in the oral cavity, and in the respiratory tract [36–39]. In addition, *Rothia* is a well-established commensal of the oral microbiome and has also been detected in indoor dust environments [40].

**Fig. 3.**
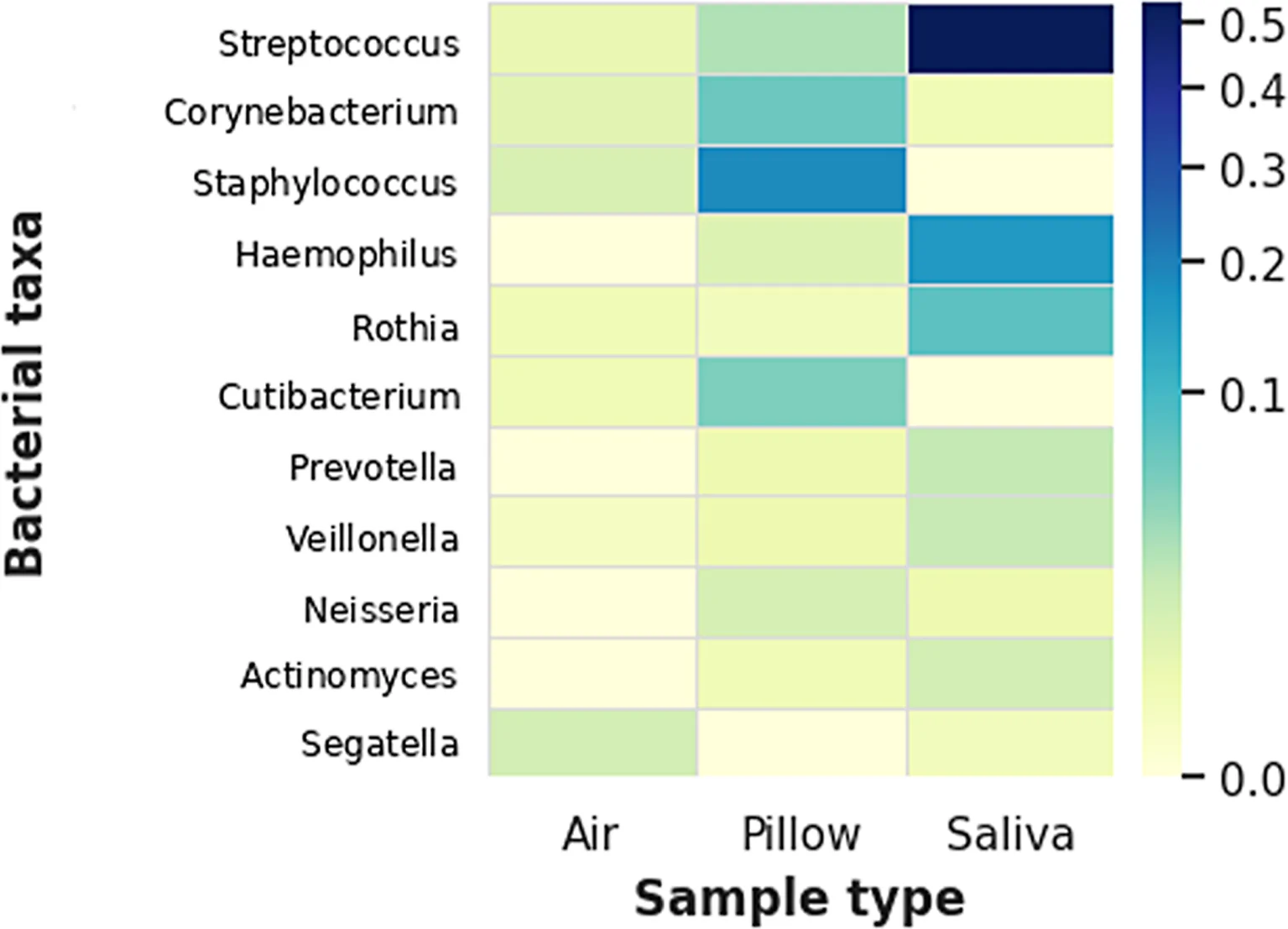
Relative abundance of dominant genera across saliva, pillow dust, and indoor air samples. Dominant genera were defined as those with a mean relative abundance of ≥1% within at least one sample type.

Environmental samples (air and pillow) showed marked inter-sample variation in microbial composition, as reflected by the large standard deviations in genus-level relative abundances and the more dispersed beta-diversity patterns. In contrast, saliva samples exhibited a more consistent microbial composition across subjects, characterized by shared core microorganisms and comparatively lower variability in dominant genera.

Microbial taxa commonly detected in pillow samples were predominantly associated with human oral, skin, and vaginal microbiota. Skin-associated genera included *Staphylococcus* spp., *Cutibacterium acnes*, and *Corynebacterium* spp. (including *C*. *tuberculostearicum)* [37]. Oral-associated taxa included *Granulicatella adiacens*, *Rothia mucilaginosa*, *Schaalia odontolytica*, several *Staphylococcus* spp., and *Granulicatella* spp. [41, 42]. In addition, *Lactobacillus iners*, a well-established vaginal commensal, was also detected in pillow samples [37].

Correlation analysis revealed significant associations between air and pillow microbiota at the genus level. *Brevitalea*, *Paludibaculum*, *Gardnerella*, *Lactobacillus*, *Faecalibacterium*, and *Segatella* showed significant correlations (*p* < 0.05), with correlation coefficients of 0.96, 0.91, 0.99, 0.73, 0.83, and 0.90, respectively. Between pillow and saliva samples, *Actinomyces*, *Bifidobacterium*, and *Fusobacterium* were significantly correlated (*p* < 0.05), with correlation coefficients of 0.97, 0.99, and 0.82, respectively. At the species level, *Actinomyces oris*, *Veillonella massiliensis*, *Streptococcus gordonii*, *Campylobacter showae*, and *Veillonella atypica* showed significant correlations between pillow and saliva samples (*p* < 0.05). In contrast, *Peptostreptococcus* was the only taxon showing a significant difference between air and saliva samples.

These results suggest potential microbial exchange between indoor air and pillow surfaces, consistent with previous evidence that airborne bacteria can deposit onto indoor surfaces through gravitational settling [40]. Furthermore, the observed associations between pillow and saliva microbiota imply possible interactions between the sleep environment and the oral microbiome.

Our observations of human-associated taxa in pillow dust and bedroom air are broadly in line with previous studies showing that indoor dust microbiomes are strongly shaped by human occupancy and activity [22]. For example, work on mattress and floor dust has demonstrated that a large fraction of bacterial sequences in house dust originate from human sources such as skin and mucosal surfaces, supporting the idea of bidirectional transfer between occupants and their sleeping environments [43]. Studies of bed dust and household dust have likewise reported high bacterial loads, enrichment of gram-positive taxa including *Staphylococcus* spp., and site-specific community structures that reflect contact with the human body and resuspension from bedding materials [44]. Together with evidence that sleep alters oral microbial communities and that oral microbiome diversity is linked to sleep duration, these findings suggest that the sleep period may be an important window for human–environment microbial exchange, with pillow dust and bedroom air acting as intermediary reservoirs that interact with the oral cavity [45]. Our results extend prior indoor microbiome and dust studies by focusing specifically on the bedroom microenvironment and oral microbiota, highlighting a potentially underappreciated axis of human–environment microbial transfer during sleep.

However, due to the limited sample size, statistical significance was not retained when samples from a single subject were excluded. This was a pilot study with a small sample size, and at least one sample was excluded because of insufficient DNA for sequencing. Some correlations were, therefore, sensitive to the inclusion of individual participants and lost statistical significance when a single subject was removed, indicating that these associations should be interpreted as exploratory and hypothesis-generating rather than conclusive. In addition, contextual information on household and sleep-related behaviors was not systematically collected, which limits our ability to fully interpret the presence of vaginal-associated taxa such as *L. iners* in pillow dust. Therefore, while this study suggests potential relationships among air, pillow, and saliva microbiomes in the sleep environment, caution is warranted when inferring their direct effects. Future studies with larger, adequately powered cohorts (on the order of several dozen participants) will be required to confirm these associations, to more precisely quantify host-environment microbiome relationships in the sleep environment, and to further elucidate the links between environmental exposures and the oral microbiome. Moreover, we did not profile other body sites such as the nasal cavity or skin, which are in direct contact with the bedroom air and pillow surface; future studies integrating nasal and skin microbiomes with saliva, pillow dust, and indoor air will be important to more fully resolve host-environment microbial exchange during sleep.

In conclusion, this tri-source dataset offers a unique opportunity to explore potential host-environment microbial relationships during rest. Pillow, air, and saliva microbiomes were compositionally distinct yet partially overlapping, suggesting possible microbial links between the human host and the surrounding built environment. The sleep environment, represented by pillow dust and bedroom air, may, therefore, constitute an important component of the exposome that is associated with the oral microbiome, although our cross-sectional, DNA-based data do not allow causal inferences about viability or temporal dynamics. These findings highlight the sleep environment as a relevant exposomic factor and provide a basis for future longitudinal and mechanistic studies.

## Acknowledgments

The authors thank all participants for their involvement in this study and acknowledge the support of the research staff, including JH Seo, for their contributions to sample processing and data analysis.

## Statement of Ethics

This study was approved by the Institutional Review Board of Konkuk University Medical Center (IRB Approval No. 2024-09–028). Written informed consent was obtained from all participants prior to participation after a detailed explanation of the study objectives and sampling procedures.

## Conflict of Interest Statement

The authors have no conflicts of interest to declare.

## Funding Sources

This study was supported by the Semyung University Research Grant of 2025. The funding source had no role in study design; data collection, analysis, or interpretation; manuscript conception, writing, or revision; or the decision to submit the manuscript for publication.

## Author Contributions

Clinical sampling: Jang JY and Lee J. Environmental sample collection: Jang JY and Yang J. Investigation: Jang JY, Lee J, Yun H, and Yang J. Data analysis and visualization: Yun H and Yang J. Conceptualization, supervision, and writing – review and editing: Yang J. Writing – original draft: Jang JY.

## Data Availability

The datasets generated during the current study are available in the Sequence Read Archive (SRA) repository, PRJNA1250390. Further enquiries can be directed to the corresponding author.
